# In Vitro Bioaccessibility of Ripe Table Olive Mineral Nutrients

**DOI:** 10.3390/foods9030275

**Published:** 2020-03-03

**Authors:** Antonio López-López, José María Moreno-Baquero, Antonio Garrido-Fernández

**Affiliations:** Food Biotechnology Department, Instituto de la Grasa (CSIC), Campus Universitario Pablo de Olavide, Edificio 46, Ctra. Utrera km 1, 41013 Sevilla, Spain; jose.moreno.baquero@gmail.com (J.M.M.-B.); garfer@cica.es (A.G.-F.)

**Keywords:** sodium, potassium, calcium, magnesium, iron, phosphorus, darkened by oxidation olives, Miller’s protocol, Crews’ protocol, post-digest re-extraction

## Abstract

For the first time, the bioaccessibility of the mineral nutrients in ripe table olives and their contributions to the recommended daily intake (RDI), according to digestion methods (Miller’s vs. Crews’ protocols), digestion type (standard vs. modified, standard plus a post-digest re-extraction), and mineralisation system (wet vs. ashing) were studied. Overall, when the standard application was used, Miller’s protocol resulted in higher bioaccessibilities of Na, K, Ca, Mg, and Fe than the Crews’ method. The modified protocols improved most of these values, but the Crews’ results only approximated the Miller’s levels in the case of Na and K. The bioaccessibility of P was hardly affected by the factors studied, except that the modified Miller’s protocol led to higher levels when ashing. No significant effect of the mineralisation system was found. The modified Miller’s protocol, regardless of the mineralisation system, led to the overall highest bioaccessibility values in ripe olives, which were: Na (96%), K (95%), Ca (20%), Mg (73%), Fe (45%), and P (60%). Their potential contributions to the RDI, based on these bioaccessibilities and 100 g olive flesh service size, were then 29, 0.5, 4, 3, 33, and 1% respectively. The investigation has led to the development of a method for assessing the bioaccessibility of the mineral nutrients not only in ripe but also in the remaining table olive presentations and opens a new research line of great interest for producing healthier products.

## 1. Introduction

The concentrations of mineral elements can be declared in the nutritional labelling of foods [1,2]. The Food and Drug Administration (FDA) and European Union (EU) standards also include recommended daily intakes for minerals. A detailed description of the individual requirements of these nutrients can likewise be found in the Dietary References Intakes Tables and Application issued by the Health and Medicine Division of the National Academies of Sciences, Engineering and Medicine [3].

Table olives are well known all over the world. The consolidated balance issued by the International Olive Council established a global production of 3.28 × 10^6^ t for the 2018/2019 season [4]. As with many other vegetables, the fruit storage/fermentation process takes place in brine with a NaCl concentration in an equilibrium ≥50 g/L [5]. As all the solutions used for processing are aqueous, marked leaching of minerals from the flesh into the brine (except for Na, which moves in the opposite direction) usually occurs [5]. As a result, the Na level increases while the contents of the other elements in the final products, despite these losses, remain moderately high. The concentrations reported in the literature depend on processing conditions, cultivars, and preparation styles and range between the following values: Na, 571–17,221 mg/kg; K, 81–1176 mg/kg; Ca, 337–850 mg/kg; Mg, 13–133 mg/kg; Fe, 4–132 mg/kg; and P, 57–118 mg/kg. [6]. The most significant differences were found among green (Spanish-style), directly brined (natural olives) and ripe olives (darkened by oxidation). However, the concentrations of minerals only provide information on their potential contributions to the diet. Assessment of their effective intake by consumers requires an estimation of their bioaccessibilities, defined as the proportions of the elements converted into soluble forms in the gastrointestinal tract [7,8].

Several methods have been proposed to assess the mineral bioaccessibility in foods. Miller’s protocol uses low amounts of food and reduced volumes of enzymatic solutions [7] and has been slightly modified by Mesias, Seiquer, and Navarro for studying the calcium bioavailability of diets rich in Maillard reaction products [8]. In contrast, the protocol developed by Crews, Burrell, and McWeeny [9,10] is characterised by the use of relatively high amounts of samples and the addition of substantial volumes of solutions (125 mL of intestinal juice) to mimic the liquids incorporated into the food during its passage through the gastrointestinal tract. Apart from these differences, both methodologies are based on a sequential enzymolysis [7,9,10].

At the moment, no information is available on the relative behaviour of both methods when applied to table olives. The high proportion of fat in these fruits [11] may require high proportions of bile salts. In addition, the abundant presence of Na might interfere in the solubilisation of the other elements or require a more intense extraction to reduce its presence in the final solid residue as much as possible. Therefore, the development of a method for studying the bioaccessibility of selected mineral nutrients adapted to the high fat and Na contents of table olives is an essential first step in any study of their actual contributions to RDI values and nutritional valorisation.

This work aimed to investigate the bioaccessibilities of mineral nutrients in table olives according to digestion methods (Miller’s vs. Crews’ protocols), digestion type (standard vs. modified, standard plus a post-digest re-extraction), and mineralisation systems (wet vs. ashing). This study may help the selection/adaptation of a protocol compatible with the high fat and Na content of these products and lead to results that approach the real bioaccessibilities of their minerals. Its development could facilitate further studies on other presentations and promote the nutritional value of table olives. Furthermore, as far as we know, this is the first time that an investigation on the bioaccessibility of mineral nutrients in fermented vegetables is carried out. Therefore, the work represents pioneer information in this field.

## 2. Materials and Methods

### 2.1. Samples and Experimental Design

**Figure 1:**
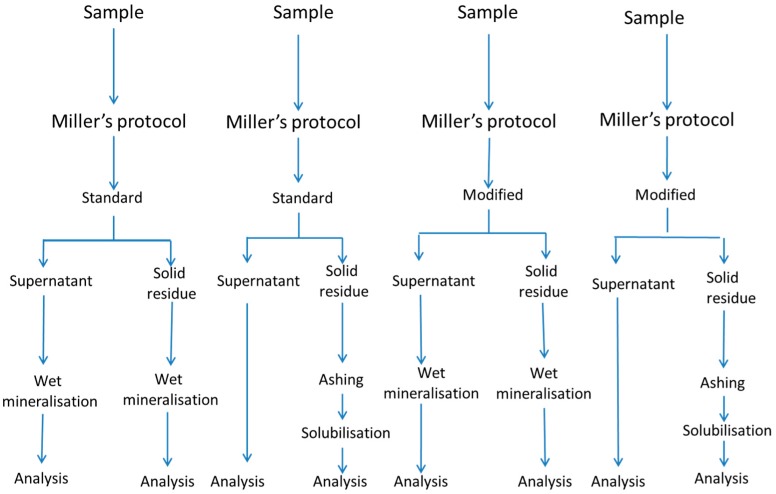
In vitro gastrointestinal digestion of ripe olives. The effect of digestion protocol (Miller vs. Crews), digestion type (standard vs. modified), and mineralisation system (wet vs. ashing) on the mineral bioaccessibility. Schema of the experimental design for Miller’s protocol. A similar one for Crews’ protocol can be obtained just by substituting Miller’s by Crews’.

Samples were of the Cacereña cultivar, picked at the green maturation stage (Caceres, Extremadura, Spain). Fruits of size 201/290 were selected and processed as ripe olives, according to the standard procedure, which consisted of three lye treatments, which progressively penetrated the flesh, followed by immersion in tap water to remove the excess alkali, and aeration. After oxidation, the olives were submerged overnight in a 0.1% ferrous lactate solution, packed in a 2.0% NaCl and 0.1% acetic acid cover brine, and sterilised at 121 °C for 45 min to reach an ${F_{0}}_{121\;{}^{\circ}C}^{10}$ (cumulative sterility value) of 15 [5]. The experiment consisted of a complete factorial design at two levels, with the variables being: gastrointestinal digestion protocol (Miller vs. Crews), digestion type (standard vs. modified, that is standard plus an additional post-digest re-extraction, using distilled-deionised water (onwards water), and mineralisation system (wet vs. ashing). Due to the impossibility of running the complete design simultaneously, each combination of variables (treatment) was carried out independently, with its raw material from the same oxidation process batch. In this way, the experiment consisted of 2^3^ different treatments, illustrated in Figure 1 for Miller’s protocol. As an example, the first treatment consisted of subjecting the olive sample to the Miller’s protocol, following the standard method, using the wet mineralisation for both the supernatant solution and the solid residue (Figure 1). All treatments were carried out in triplicate, using, for each, 100 g of homogenised olive flesh as raw material and one blank, prepared with only the reagents and run in parallel to the sample. The blank was used to evaluate the contribution of enzymes and other chemicals to the final mineral content in the digestion fractions.

The introduction of the post-digest re-extraction with water was due to the high levels of Na and K in table olives, which could hardly be solubilised in the volume of liquid used in the standard protocols. This modification may contribute to improving the solubilisation of these minerals and evaluate the strength of the complexes formed by some of them with the flesh components; furthermore, its application is in agreement with the intense nutrient exchanges between phases that take place during the gastrointestinal passage of foods and the re-extraction steps used in other works [12].

### 2.2. Cleaning of the Material

All glassware used for the determination of the minerals was immersed in 10% (*w*/*w*) nitric acid overnight and then rinsed several times with water.

### 2.3. In Vitro Digestion of Olives

#### 2.3.1. Miller’s Protocol

**Figure 2:**
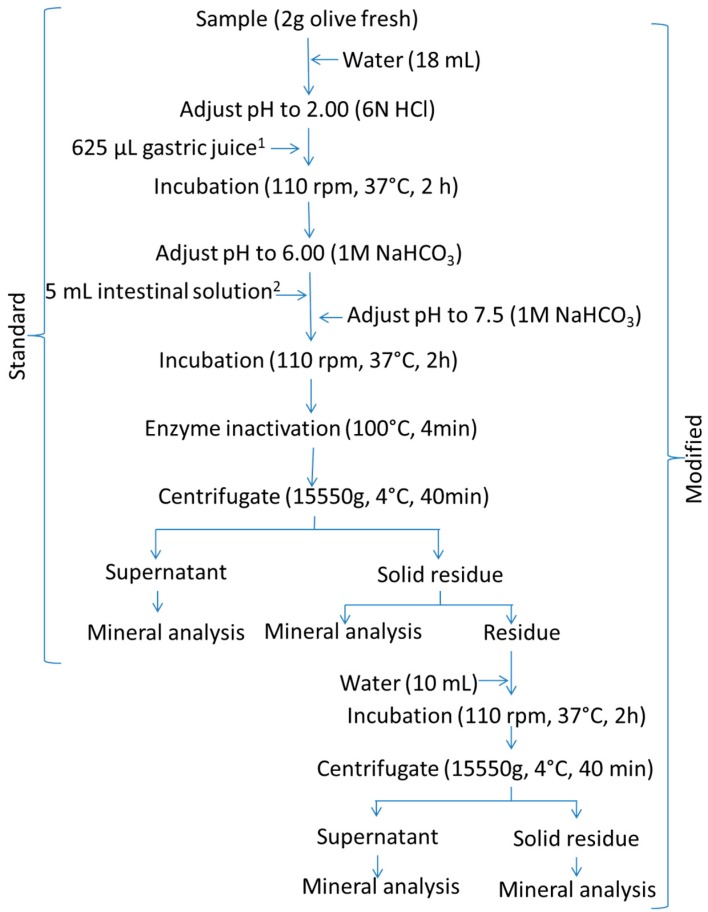
In vitro gastrointestinal digestion of ripe olives. Schema of the Miller’s standard and modified (standard plus a post-digest re-extraction) protocols. ^1^ gastric solution consisted of 0.8 g pepsin dissolved in 5 mL 0.1 N HCl. ^2^ Intestinal solution consisted of 0.1 g pancreatin and 0.625 g bile salts dissolved in 25 mL 0.1 M NaHCO_3_. Water stands for deionized-distilled water.

This method was based on Miller, Schicker, Rasmussen, and Campen [7]. A flowchart of its application is shown in Figure 2 (standard). Briefly, 2 g of homogenised olive pulp (an aliquot from the 100 g ripe olive sample) was suspended in 18 mL of water. For the gastric digestion, its pH was adjusted to 2.0 with 6 N HCl, and the mixture was added to 625 µL of simulated gastric juice (prepared by dissolving 80 mg of pepsin in 5 mL of 0.1 N HCl). The suspension was then placed in a shaking water bath incubator at 37 °C and 110 rpm for 2 h. For the intestinal digestion, the pH of the digest was raised to 6.0 with 1 M NaHCO_3_ and 5 mL of simulated intestinal juice (prepared by dissolving 10 mg of pancreatin and 62.5 mg of bile salts in 25 mL of 0.1 M NaHCO_3_) was added. The pH was then adjusted to 7.5 with 1 M NaHCO_3_, and the suspension incubated at 37 °C and 110 rpm for 2 h. After the gastrointestinal digestion, the digestive enzymes were inactivated in an oven at 100 °C for 4 min. The sample was then cooled in an ice bath and centrifuged at 15,550× *g* and 4 °C (5804R centrifuge, Eppendorf, Hamburg, Germany) for 40 min. The supernatant and the solid residue were separated and weighed, and the mineral concentration in each fraction was analysed.

#### 2.3.2. Crews’ Protocol

**Figure 3:**
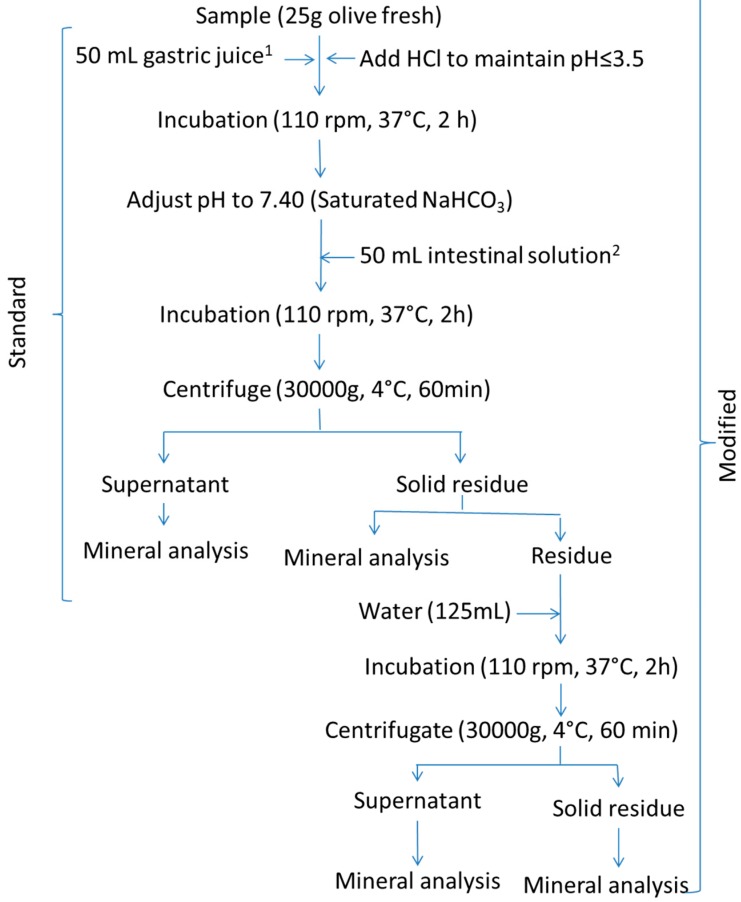
In vitro gastrointestinal digestion of ripe olives. Schema of the Crews’ standard and modified (standard plus a post-digest re-extraction) protocols. ^1^ gastric solution consisted of 1% pepsin in saline HCl (0.15 M NaCl; 0.02 M HCl) at pH 1.8. ^2^ Intestinal solution consisted of a mixture of equal volumes of (a) 3% pancreatin, 1% amylase, and (b) bile salts in saline solutions (0.15 M NaCl). Water stands for deionized-distilled water.

The procedure described in Crews, Burrell, and McWeeny [9,10] was followed (Figure 3, standard). Briefly, 25 g of homogenised olive pulp (an aliquot of the 100 g ripe olive sample) was weighed and suspended in 50 mL of simulated gastric juice, prepared by dissolving 10 mg/mL of pepsin in saline hydrochloric acid (0.15 M sodium chloride; 0.02 M hydrochloric acid) at pH 1.8. The suspension was incubated at 37 °C and 150 rpm for 2 h and 6 N HCl was added as necessary to maintain pH ≤ 3.5. After incubation, the suspension pH was adjusted to 7.4 with a saturated NaHCO_3_ solution and was added to 50 mL of simulated intestinal juice, prepared by mixing equal volumes of (a) 30 mg/mL pancreatin plus 10 mg/mL of amylase and (b) 1.5 g/L of bile salts in 0.15 M NaCl. The mixture was again incubated at 37 °C and 150 rpm for 2 h and centrifuged at 30,000× *g* and 4 °C for 60 min (Sorvall RC6 plus centrifuge, Thermo Scientific, Langenselbold, Germany). The weights and mineral concentrations in the supernatant and the solid residue were calculated as described in Miller’s protocol.

#### 2.3.3. Modified Protocols

The modification consisted of adding 10 mL, or 125 mL, of water to the digested residues from the standard Miller’s or Crews’ protocols, respectively, incubating the suspension again in a shaking water bath at 37 °C and 110 rpm for 2 h, and centrifuging at 15,000× *g* (Miller’s protocol) or 30,000× *g* (Crews’protocol) and 4 °C for 60 min (Sorvall RC6 plus centrifuge). The supernatant was combined with that from the standard protocol to form the supernatant of the modified technique. The mineral content in these supernatants and their respective re-extracted solid residues were determined. The resulting methodology will be onwards referred to as the modified protocol.

### 2.4. Mineralisation

The analysis of most fractions requires previous mineralisation. Due to the diversity of samples studied (olive paste, supernatant solutions, and post-digestion solid residues), evaluation of the effect of the mineralisation system was considered of interest. Two options were assayed.

#### 2.4.1. Wet Mineralisation

For this process, 20–25 mL of the supernatants were concentrated to 15 mL in a flask, and then added to 5 mL of 65% HNO_3_. The container was then heated in a shaking sand bath at 180–220 °C until the liquid was clear or pale straw-coloured and orange fumes ceased. Then, 5 mL of a mixture of HNO_3_ (65%)–HClO_4_ (60%) (1:4) was added, and the solution was heated at 180–220 °C until discolouration and white fumes evolved. The samples were cooled, transferred into a 25 mL volumetric flask and made up to volume with water.

For the homogenised olive flesh (raw material) and the solid residues of the digestions, 2.5 g of the paste were weighed into a 50 mL Erlenmeyer flask and added to 5 mL of HNO_3_ (65%). Then, the suspensions were subjected to the same steps described above for liquids.

#### 2.4.2. Ashing

This method was applied only to solids as the solutions were directly submitted to the analysis. In short, 2.5 g of sample (homogenised olive flesh or solid residues from the digestions) was weighed in a quartz capsule and placed in a muffle oven whose temperature was quickly brought to 100 °C, followed by a slow increase up to 550 °C. After incineration for 8–10 h, the ashes, greyish-white in colour, were moistened and dissolved (slightly warming the capsule) in three portions of 2 mL 6 N HCl and filtered through a filter paper into a 25 mL volumetric flask, using a suction hood. After washing the filter three times with 3 mL of water, the solution was made up to volume with water.

### 2.5. Mineral Analysis

Na, K, Ca, Mg, and Fe were analysed by atomic absorption spectrophotometry, using an air-acetylene flame and the analytical conditions were recommended by the equipment manufacturer [13]. The value for each triplicate was the average of three determinations.

To prevent interferences and ionisation of the air-acetylene flame, the aliquots for analysis and the calibration standards were added to lantane (0.5%, *w*/*v*), when analysing Ca and Mg, or potassium (0.1%, *w*/*v*) and sodium (0.1%, *w*/*v*), in the case of Na and K, respectively.

Phosphorus was analysed following the official method of the AOAC n° 970-39 Phosphorus in Fruits and Fruit Products (spectrophotometric molybdovanadate method) [14]. This method is based on the absorbance at 400 nm of the yellow phospho-molybdovanadate complex formed in the presence of V^5+^ and Mo^6+^. The value for each triplicate was also the average of three determinations.

Calibration curves were obtained daily from successive dilutions of the stock solutions. Interpolation was always made after subtracting the signal of the blank from those of the samples. Furthermore, samples of standard solutions were also periodically included in the determinations.

### 2.6. Apparatus and Reagents

The equipment included a GBC model 932 AA (GBC, Braeside, VIC, Australia) atomic absorption spectrometer equipped with three hollow multi-element cathode lamps, (Na and K) (Photron, Narre Warren, VIC, Australia), (Cu, Fe, and Mn) (GBC, Braeside, VIC, Australia) and (Ca, Mg, Cu, and Zn) (Photron, Narre Warren, VIC, Australia); a Cary UV/Visible spectrophotometer model 1E (Varian Australia, Mulgrave, Victoria); a shaking water bath incubator (WY-200 COD. 5312091, COMECTA, S.A., Barcelona, Spain); and a shaking sand bath incubator (Combiplac-Sand 6000709; J.P. Selecta, Barcelona, Spain).

All reagents were of analytical grade. The enzymes and bile salts were purchased from Sigma-Aldrich (pepsin from porcine gastric mucosa Cat N° P7000; pancreatin from porcine pancreas Cat N° P1750; α-amylase from porcine pancreas Cat N° A3176; and bile salts Cat N° B8756).

### 2.7. Mineral Recovery and Bioaccessibility Estimation

The calculous were carried out independently for each treatment (the combination of factors). Considering the concentrations in the raw material, the supernatant solutions (including that from the modified protocols), the solid residue, and the blank, the amount of each mineral nutrient in them (RM, S, SR, and B, respectively) were estimated. The amount of mineral in each of these fractions was calculated taking into account the weight of each of them, which allows a correct mass balance. Bioaccessibility and recovery (expressed as percentages) were estimated using the following formulae:

(1) $$\mathrm{Bioaccessibility}\;\left(\%\right)=\left(\frac{\left(S-B\right)}{\mathrm{RM}}\right)\times 100$$

(2) $$\mathrm{Recovery}\;\left(\%\right)=\left(\frac{\left(S-B+\mathrm{SR}\right)}{\mathrm{RM}}\right)\times 100$$

An approach to the contribution of the ripe olives to the RDI of the minerals studied (Na, K, Ca, Mg, Fe and P), based on their bioaccessible amount in 100 g olive flesh serving size, were also deduced.

### 2.8. Statistical Analysis

The effect of the different factors (digestion protocols, digestion type, and mineralisation system) on bioaccessibility was studied by General Linear Model (GLM). The effects were considered significant at *p* ≤ 0.05 when the corresponding confidence limits (CL) of their averages did not overlap. The study was carried out using Statistic v. 8.0 (StatSoft, Inc., Tulsa, OK, USA) [15].

## 3. Results and Discussion

Through processing, ripe olives are usually in contact with solutions containing Na (brine and lye), Ca (mainly during storage and final packaging), and Fe (for fixing the colour) [5], which increase their contents in the flesh. The concentrations of these minerals in the different samples used as raw material were high. The Na ranged from 7085 to 7181 mg/kg (Table 1 and Table 2), lower than the levels reported for any other table olive presentation [6]. The Ca content, 1666–1711 mg/kg (Table 1 and Table 2), was higher than in green plain Spanish-style or directly brined olives [6]. However, the most notable difference was found for Fe because its content was particularly high (102 to 105 mg/kg) with respect to any other non-oxidized olive product (3.49–7.70 mg/kg) [6].

Regarding other mineral nutrients not intentionally incorporated during processing, their contents suffer a progressive diminution during the elaboration [5]. However, the ripe olives of this experiment still retained substantial levels of Mg (129 to 138 mg/kg), K (104 to 109 mg/kg), and P (91 to 100 mg/kg) (Table 1 and Table 2).

The differences among the mineral contents in diverse raw materials (ripe olive samples) were relatively close since they came from the same batch; but, even in such circumstances, the use of a specific raw material for each treatment (a combination of factors) was considered convenient to eliminate this source of variability on bioaccessibility.

Most of the enzymes and bile salts used for the digestion also contained nutrient elements that contributed to the mineral levels in the final fractions (see contents in the blanks) (Table 1 and Table 2). The levels were particularly high in Na, whose concentrations ranged from 1002 to 1309 mg/kg in Miller’s protocol but was markedly higher (5008–6139 mg/kg) in Crews’ method. Furthermore, the levels of P (25.9–28.1 mg/kg, Miller; 37.7–38.8 mg/kg, Crews) and K (18.4–20.3 mg/kg, Miller; 31.3–33.4 mg/kg, Crews) were also relevant, but not the presence of the other nutrients, which were low (Table 1 and Table 2). In any case, the concentrations in the supernatants were always corrected by subtracting the corresponding blanks.

### 3.1. Mineral Linkage to Olive Flesh Components and Post-Digestion Extraction

The response of the mineral nutrients in foods to digestion is strongly related to their aqueous solubility and linkage to the structural components of the products. According to the literature [16], the bioaccessibility of Na is usually considered complete but, due to the high salt concentration in table olives, its response could not be straightforward but requires investigation.

In the standard protocols, the concentration of Na in the solid residue was higher than in the supernatant solution (average 2413 vs. 1806 mg/kg for Miller; average 7544 vs. 6108 mg/kg for Crews), regardless of the mineralisation system (Table 1 and Table 2). However, applying modified protocols, the Na concentrations in both fractions reversed, being markedly higher in the supernatant fractions than in the solid residues (average 1221 vs. 282 mg/kg, Miller; average 3596 vs. 966 mg/kg, Crews) (Table 1 and Table 2). Hence, the application of one post-digest re-extraction to the standard protocols constitutes a closer approach to reality [9,10]. Furthermore, this also means that Na is weakly linked to the ripe olive flesh components.

The distributions of K in the two fractions of the digestion (standard or modified) followed a similar trend to Na, regardless of treatments (Table 1 and Table 2). This behaviour may also indicate that K could be, in practice, completely bioaccessible when subjected to the conditions prevailing in the gastrointestinal tract [16] and that it is weakly retained in the ripe olive flesh.

On the contrary, the concentrations of Ca in the supernatant solutions after digestion were very low with respect to the solid fraction, regardless of the technique applied (Table 1 and Table 2) Furthermore, when the modified protocol was applied, the release was not improved despite the still high Ca levels in solid residues (Table 1 and Table 2). In consequence, no equilibrium between solid residues and supernatant solutions could be expected even in the case of a large number of post-digest re-extractions. Such behaviour means a strong Ca linkage to the olive flesh components, resistant to the digestive enzymes. These data are in agreement with the literature reports on Ca absorption by olives [17]. Furthermore, this flesh capacity for Ca bounding is used for improving texture during green olive fermentation and ripe olive storage [5].

Magnesium is not added during processing; on the contrary, leakage through elaboration is common [5]. After standard digestion, its concentrations in the supernatant solutions (Table 1 and Table 2) were markedly lower than the levels in the solid residues, regardless of digestion technique, following, in this case, a similar trend to Na, K or even Ca. However, applying post-digest re-extraction, the contents in the solid residues remained similar (Miller) or slightly decreased (Crews). Therefore, its behaviour in the modified protocol was completely different from that followed by Na and K but approached that of Ca, with a higher solubilisation. Therefore, Mg was retained in the olive flesh more than Na and K but less than Ca, meaning that at least part of it can also be bound to the ripe olive flesh.

As inferred from the low concentrations of iron found in the standard and modified protocol supernatants and its high contents in the solid residues, strong retention of iron by the ripe olive flesh is evident (Table 1 and Table 2). This behaviour is somewhat similar to that of Ca, although may have a different origin since such absorption has been related to the formation of complexes between this element and polymers from hydroxytyrosol and caffeic acid, which are produced during the ripe olive darkening (oxidation) process [5].

Due to the relatively high content of P (not added during elaboration) in the raw material, it is evident that there is a strong link between this element and the olive flesh, which has resisted the successive alkali treatment and tap washings applied throughout processing [5]. The enzymes used for the digestion produced a marked solubilisation of P (Table 1 and Table 2), although still left a sensible proportion of it in the solid residue, which was not solubilised by the post-digest re-extraction. Therefore, P was relatively resistant to the digestion attack but weaker than that observed for Ca or Fe, stronger than Na and K, and slightly weaker than Mg.

Hence, the application of a post-digestion extraction was useful not only for a more exhaustive removal of some minerals (Na and K) from the solid residue but also for assessing the different linkage degrees of the studied minerals and the olive flesh components. The results from the modified protocol re-affirm the hypothesis that the resistance to solubilisation of Ca, Fe (mainly) or Mg and P (in lower proportions) was not just a matter of equilibrium between phases but was also related to their bounding strength to the olive flesh. Crews, Burrell, and McWeeny [9,10] also observed similar behaviour for some of these nutrients in the cereal food group [12].

### 3.2. Effect of Diverse Factors on the Mineral Bioaccessibility

**Figure 4:**
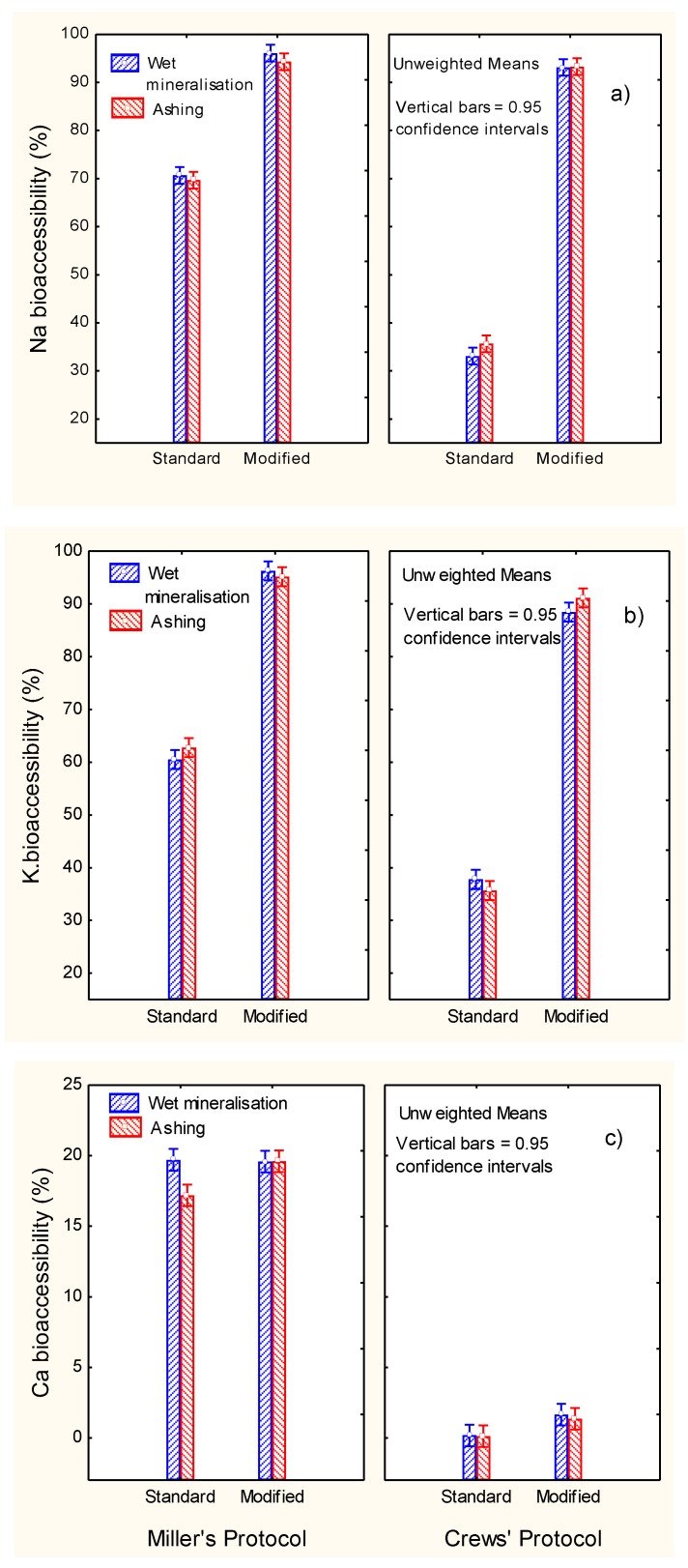
In vitro gastrointestinal digestion of ripe olives. Effect of digestion protocol (Miller vs. Crews), and digestion type (standard vs. modified) and mineralisation system (wet vs. ashing) on bioaccessibility (%) of (**a**) Na, (**b**) K and (**c**) Ca.

The weights of the raw materials, blank solutions and the different digestion fractions required for evaluating the mineral recovery are shown in Table 3. The markedly higher weights of olive samples and digested fractions in the Crews’ technique are apparent. When applying the modified protocols, the weights of supernatants are the sum of those from the standard supernatant solutions of enzymes plus those from the post-digest re-extraction. From the data in Table 1, Table 2 and Table 3, the bioaccessibility of the minerals were estimated (Equation (1)). Overall, the mineralisation system had scarce or no effect on the bioaccessibility results. Regarding digestion methods, the bioaccessibilities of Na and K (Figure 4a,b) were markedly higher in Miller’s than in Crews’ standard protocols, but the application of the modified protocol considerably increased them to values greater than 90% (mainly in Crews’ methodology), with only slight differences between protocols in the case of Na and a significant difference in favour of Miller’s for K. Hence, the notable increase in the recuperation of these elements with the post-digest re-extraction means that they could be completely bioaccessible from ripe olives (progressive dilution and absorption from the human gut), as confirmed by their weak interaction with the flesh components and as already suggested in the literature for other foods [9,10].

In contrast, the bioaccessibility of Ca was always low (Figure 4c), although it was higher when applying Miller’s protocol. However, the use of the modified protocols hardly led to any further improvement. As a result, the potential contribution of ripe olives, and possibly other presentations, to Ca in the diet could be minimal, despite the relatively high proportion of this element usually present in the product. However, this problem is not exclusive of olives because low bioaccessibility levels of Ca have also been observed in other foods, such as school meals (0.75%), with the lowest bioaccessibility found in vegetables [18] or milk, where calcium was partially soluble and ranged from 48% to 62% [19].

**Figure 5:**
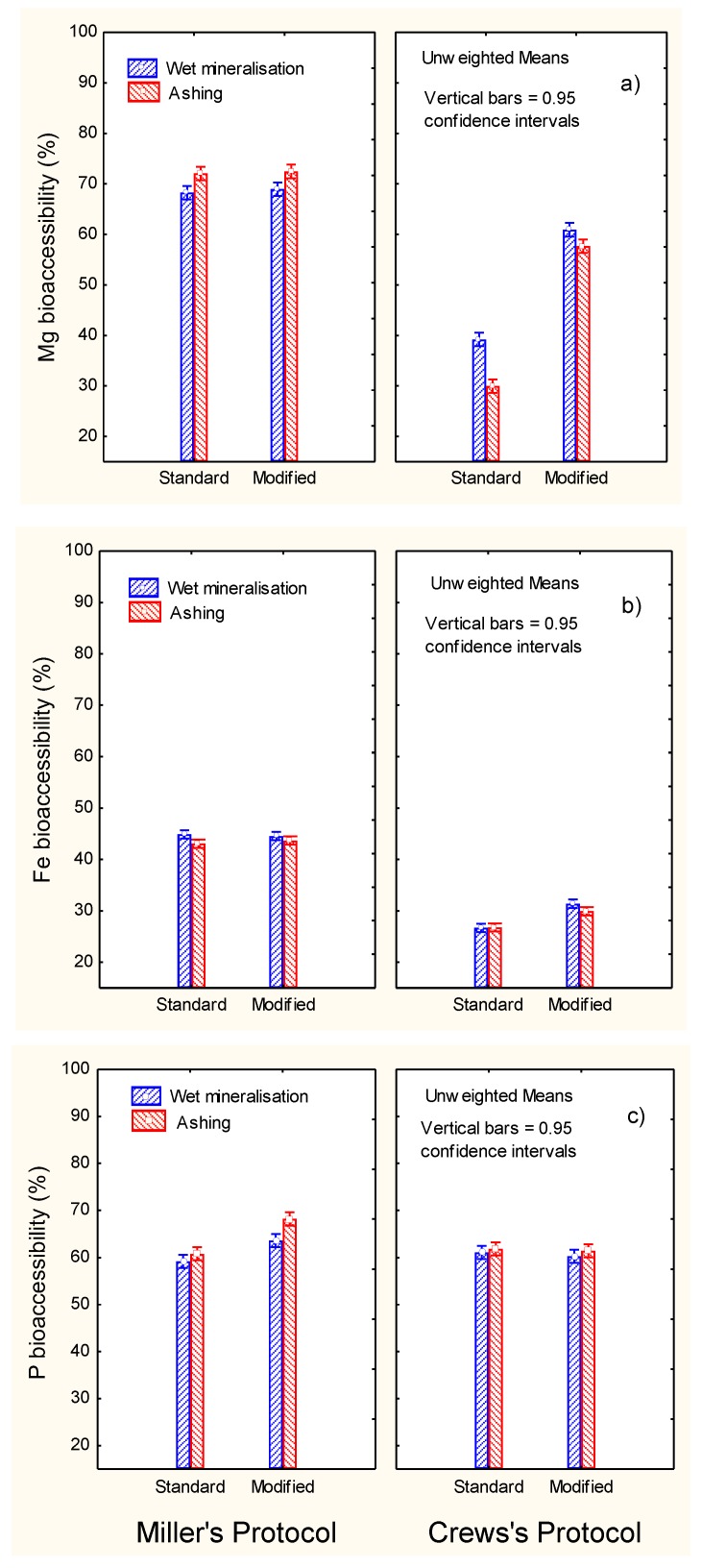
In vitro gastrointestinal digestion of ripe olives. Effect of digestion protocol (Miller vs. Crews), and digestion type (standard vs. modified) and mineralisation system (wet vs. ashing) on bioaccessibility (%) of (**a**) Mg, (**b**) Fe, and (**c**) P.

The highest bioaccessibility of Mg (Figure 5a) was observed using the standard Miller’s protocol (above 70%) without any effect of post-digest re-extraction. The modified Crews’ protocol increased the bioaccessibility versus the standard but without reaching Miller’s levels. Therefore, Crews’ conditions were scarcely efficient for studying Mg bioaccessibility. According to the literature, Mg bioaccessibility may be influenced by the compound used in the diet; the ingestion of citrate was more efficient that oxide [20]. Mg absorption in healthy women is reported to be incremental (11–14%) when mineral water was consumed alone or in combination with meals [21]. Furthermore, the mineralisation level (sulfate, bicarbonate, or calcium) in mineral waters did not influence the Mg bioavailability [22].

The highest bioaccessibility of Fe (approx. 45%) was observed when applying Miller’s protocols (standard or modified) (Figure 5b). The re-extraction had a limited effect on the Crews’protocol, changing from approx. 27% (standard) to just above 30% (modified). As in the case of Ca and Mg, Miller’s protocol was more efficient (approx. 45% bioaccessibility) and reached an intermediate level between them (approx. 20 and 70%, respectively). In previous studies by Crews, Burrell, and McWeeny [12], high percentages of iron solubility were reported; about 25% (cereals) and 75% (vegetables). The percentage solubility of iron in whole-meal bread was approx. 35%, while in crab it was sensibly lower (5%) [9]. In selected foods (meat as well as bread, milk, and beverage substitutes), the iron available was in general low (below 10%), reaching only slightly higher proportions in orange juice (about 25%) and cheese plus orange juice (about 17%) [7]. Therefore, iron bioaccessibility in ripe olives could be higher than in more common foods. Its low bioaccessibility has been related to the presence of oxalic acid and egg proteins, while meat had a favourable effect [18].

Phosphorus bioaccessibilities (Figure 5c) were relatively high (about 60%) and improved (5–10%) when applying the post-digest re-extraction only in the case of Miller’s protocol. A study of the in vitro P digestible in meat and milk products showed better absorbability in foods of animal origin than, for example, legumes [23]. However, legumes may be a relatively poor source of P, while in products containing phosphates additives, the digestible P was easily available [24]. In general, P from plants is not well absorbed because this element is stored in the form of phytic acid or phytate, which may interfere with its absorption [25]. The relative high bioaccessibility of P in table olives may be related to the low/absence presence of phytate.

### 3.3. Mineral Recovery during Digestion

The data in Table 1, Table 2 and Table 3 allow for a complete estimation of the mineral recovery, regardless of the supernatant type and solid residue. The comparison of their sum with the weights of the minerals initially present in the samples is straightforward (Equation (2)). The results show good overall recovery for all the mineral nutrients analysed (Table 4).

### 3.4. Contribution of Ripe Olives to Daily Recommended Mineral Intake

The ripe olive minerals’ bioaccessibilities can be used for estimating the potential contribution of the product to their RDI [2] (Table 5). Their values are affected by the three factors involved in the experiment, similar to their bioaccessibilities (they are just linear combinations of these), and do not require further comments. Overall, Miller’s protocol led to higher contributions. To emphasise that the ripe olives have an outstanding contribution to the RDI of Fe in the diet, which reaches about 34%, far above the 15% limit required to be considered as a significant source of this mineral and be declared in the label. Furthermore, its impact is higher than that of Na (approx. 28%).

## 4. Conclusions

The digestion protocols had significant effects on the bioaccessibility estimation of ripe olive mineral nutrients. Overall, Miller’s protocol led to higher values than Crews’ protocol. The application of a post-digest re-extraction improved (vs. standard digestion) the potential bioaccessibility of the most soluble minerals. The application of this modification was useful to evaluate the strength of the linkage between some elements and olive flesh components. Monovalent minerals (Na and K) were hardly bound and were completely bioaccessible. In contrast, the noticeable presence of divalent (and P) elements in the final solid residue indicated that at least some of them can still be strongly linked to olive flesh even after digestion. Among these cations, Ca was the most vigorously retained and, as a result, showed the lowest bioaccessibility (a maximum of approx. 20%); Mg was weakly bound and showed a high bioaccessibility level (>70%). P reached intermediate values of 60–70%. Fe was moderately retained and showed a bioaccessibility of about 45%. Based on these data, the contribution of 100 g ripe olive flesh to RDI of Fe can be estimated as approx. 34%, which allowed consideration of the product as a source of this element while maintaining a moderate Na level (about 28%) and a negligible impact of the other elements.

The modified Miller’s protocol, which includes a post-digest re-extraction, uses less sample, produces a lower volume of supernatant solutions (and solid residues), and, overall, leads to higher bioaccessibility values; therefore, it is proposed for further studies on the bioaccessibility of mineral nutrients in table olives in general.

## Figures and Tables

**Table 1:** In vitro gastrointestinal digestion of ripe olives, using the Miller’s protocol. Effect of the digestion type (standard vs. modified) and mineralisation system (wet vs. ashing) on the mineral concentrations in the supernatants and solid residues. The contents in the raw material (samples) and blanks are also provided since they are required for the estimations of mineral recoveries and bioaccessibilities. Concentrations in mg/kg.

		Standard				Modified			
Element	Mineralisation	Raw Material	Supernatant Solution	Solid Olive Residue	Blank	Raw Material	Supernatant Solution	Solid Olive Residue	Blank
Na	Wet	7132 (20)	1861 (8)	2089 (110)	1309 (8)	7104 (30)	1184 (1)	292 (14)	1002 (5)
	Ashing	7181 (5)	1752 (5)	2738 (89)	1215 (6)	7085 (11)	1257 (11)	272 (9)	1111 (6)
K	Wet	109.4 (0.7)	28.0 (0.1)	41.0 (1.7)	20.3 (0.4)	104.8 (1.0)	20.2 (<0.1)	5.2 (0.4)	18.4 (<0.1)
	Ashing	107.4 (1.0)	26.9 (<0.1)	55.1 (3.1)	19.4 (0.2)	107.0 (1.5)	21.2 (<0.1)	4.8 (0.1)	19.6 (<0.1)
Ca	Wet	1689.0 (8.0)	29.7 (0.6)	1275.7 (66.4)	1.7 (0.0)	1666.2 (3.6)	20.7 (0.1)	1449.9 (63.8)	1.4 (<0.1)
	Ashing	1700.6 (6.0)	29.5 (0.2)	1751.5 (104.4)	1.9 (<0.1)	1698.4 (6.0)	21.0 (0.1)	1713.4 (4.1)	1.7 (<0.1)
Mg	Wet	133.4 (0.4)	12.4 (<0.1)	38.3 (2.0)	3.9 (<0.1)	138.4 (0.2)	9.01 (<0.1)	45.0 (1.6)	3.9 (<0.1)
	Ashing	129.7 (0.9)	12.1 (<0.1)	48.9 (2.7)	3.8 (<0.1)	128.7 (0.8)	8.4 (<0.1)	50.1 (0.8)	3.7 (<0.1)
Fe	Wet	102.6 (0.8)	4.8 (<0.1)	55.7 (2.5)	0.9 (<0.1)	101.8 (0.9)	3.0 (<0.1)	66 (3.7)	0.4 (<0.1)
	Ashing	104.5 (1.1)	4.5 (<0.1)	77.6 (5.4)	0.6 (<0.1)	104.6 (2.4)	3.4 (<0.1)	77.0 (0.6)	0.7 (<0.1)
P	Wet	94.3 (0.4)	33.2 (0.1)	34.7 (1.0)	25.9 (<0.1)	91.3 (1.4)	25.1 (<0.1)	38.2 (1.3)	28.1 (<0.1)
	Ashing	94.8 (0.6)	NA	48.6 (2.7)	NA	99.8 (0.8)	NA	48.4 (0.5)	NA

Average of three independent experiments; standard error in parentheses; NA, not available.

**Table 2:** In vitro gastrointestinal digestion of ripe olives, using the Crews’ protocol. Effect of the digestion type (standard vs. modified) and mineralisation system (wet vs. ashing) on the mineral concentrations in the supernatants and solid residues. The contents in the raw material (samples) and blanks are also provided since they are required for the estimations of mineral recoveries and bioaccessibilities. Concentrations in mg/kg.

		Standard				Modified			
Element	Mineralisation	Raw Material	Supernatant Solution	Solid Olive Residue	Blank	Raw Material	Supernatant Solution	Solid Olive Residue	Blank
Na	Wet	7120 (7)	6139 (63)	7940 (220)	6139 (63)	7121 (25)	3653 (11)	981 (20)	5008 (11)
	Ashing	7116 (5)	6076 (85)	7148 (27)	5100 (37)	7157 (12)	3539 (21)	950 (30)	5098 (13)
K	Wet	104.0 (0.8)	44.7 (0.4)	112.1 (2.0)	33.4 (0.2)	107.6 (0.3)	28.6 (0.1)	19.0 (1.2)	31.3 (0.1)
	Ashing	105.5 (1.6)	42.8 (0.6)	103.2 (2.0)	32.1 (<0.1)	105.0 (0.7)	27.2 (0.1)	16.2 (0.2)	31.3 (0.2)
Ca	Wet	1710.5 (8.3)	1.3 (<0.1)	2949.3 (56.8)	0.6 (<0.1)	1693.9 (4.6)	3.4 (<0.1)	2686.0 (4.6)	0.6 (<0.1)
	Ashing	1702.6 (7.3)	1.2 (<0.1)	2585.0 (26.7)	0.6 (<0.1)	1709.9 (8.3)	3.3 (<0.1)	2580.7 (120.1)	0.5 (<0.01)
Mg	Wet	132.0 (2.1)	15.0 (<0.1)	163.3 (5.3)	6.1 (<0.1)	132.6 (1.0)	11.6 (<0.1)	82.4 (3.3)	5.3 (<0.1)
	Ashing	134.5 (0.7)	14.3 (0.2)	143.0 (1.0)	5.6 (<0.1)	133.5 (1.5)	11.8 (<0.1)	79.3 (2.9)	6.1 (<0.1)
Fe	Wet	103.3 (2.1)	6.0 (<0.1)	131.0 (4.5)	0.35 (<0.1)	103.5 (0.7)	3.7 (<0.1)	125.1 (11.9)	0.7 (<0.1)
	Ashing	104.6 (1.6)	6.3 (0.1)	119.6 (2.3)	0.8 (<0.1)	103.7 (1.0)	3.7 (<0.1)	114.1 (4.7)	0.6 (<0.1)
P	Wet	96.2 (0.8)	53.6 (0.5)	69.5 (0.7)	37.7 (0.2)	96.1 (0.4)	29.1 (0.1)	59.9 (0.9)	38.8 (0.1)
	Ashing	94.7 (1.1)	NA	57.2 (0.7)	NA	95.4 (1.6)	NA	59.0 (1.9)	NA

Average of three independent experiments; standard error in parentheses; NA, not available.

**Table 3:** In vitro gastrointestinal digestion of ripe olives. Weights of the raw materials (samples), the different fractions obtained after digestion, and the blank solutions, according to digestion protocols (Miller vs. Crews), digestion type (standard vs. modified), and mineralisation system (wet vs. ashing). The information allows estimation of the mineral recovery and bioaccessibility. Data are expressed in g.

Technique	Type of Digestion	Mineralisation	Sample Weight	Supernatant Solution	Solid Residue	Blank Solution
Miller	Standard	Wet	2.011 (0.004)	24.020 (0.096)	2.163 (0.094)	26.406 (−)
		Ashing	2.030 (0.012)	24.070 (0.086)	1.599 (0.121)	26.345 (−)
	Modified	Wet	2.022 (0.002)	34.005 (0.088)	1.849 (0.086)	26.454 (−)
		Ashing	2.041 (0.009)	34.218 (0.109)	1.622 (0.021)	26.372 (−)
Crews	Standard	Wet	25.060 (0.025)	123.160 (1.194)	14.533 (0.405)	136.530 (−)
		Ashing	25.017 (0.038)	125.802 (1.931)	16.272 (0.265)	138.090 (−)
	Modified	Wet	25.170 (0.067)	236.286 (0.850)	15.753 (0.469)	139.150 (−)
		Ashing	25.407 (0.194)	244.145 (1.659)	16.424 (0.594)	136.510 (−)

Average of three independent experiments; standard error in parentheses. Each digestion had its blank.

**Table 4:** In vitro gastrointestinal digestion of ripe olives. Overall mineral recovery (expressed as %), according to digestion protocols (Miller vs. Crews), digestion type (standard vs. modified), and mineralisation system (wet vs. ashing).

Technique	Type of Digestion	Mineralisation	Na	K	Ca	Mg	Fe	P
Miller	Standard	Wet	102.20 (0.26)	101.32 (0.36)	100.61 (0.48)	103.30 (0.34)	102.45 (0.28)	99.45 (0.49)
		Ashing	99.84 (0.52)	102.43 (0.33)	99.90 (0.42)	102.32 (0.24)	102.17 (0.24)	100.00 (NA) *
	Modified	Wet	99.59 (0.58)	99.65 (0.28)	99.02 (0.38)	102.11 (0.23)	104.03 (0.24)	98.33 (0.42)
		Ashing	97.90 (0.31)	99.70 (0.52)	99.86 (0.55)	103.41 (0.17)	103.74 (0.14)	100.00 (NA) *
Crews	Standard	Wet	97.98 (0.35)	98.95 (0.62)	100.06 (0.49)	101.99 (0.48)	100.16 (0.56)	102.17 (0.40)
		Ashing	98.95 (0.74)	99.95 (0.68)	98.91 (0.59)	99.54 (0.43)	101.84 (0.79)	100.00 (NA) *
	Modified	Wet	101.30 (0.49)	99.02 (0.26)	100.65 (0.35)	98.27 (0.28)	100.03 (0.54)	99.80 (0.50)
		Ashing	100.94 (0.49)	99.08 (0.38)	98.93 (0.42)	98.91 (0.38)	101.87 (0.28)	100.00 (NA) *

n = 3 for each treatment; * bioaccessibility estimated by difference.

**Table 5:** Contribution (expressed as %) of ripe olives to the RDI of the nutrient mineral studied, according to digestion protocols (Miller vs. Crews), digestion type (standard vs. modified), and mineralisation system (wet vs. ashing). Data are based on their bioaccessibilities and 100 g olive flesh.

Technique	Type of Digestion	Mineralisation	Na	K	Ca	Mg	Fe	P
Miller	Standard	Wet	21.04 (0.08)	0.336 (0.001)	4.16 (0.06)	1.09 (0.01)	32.40 (0.14)	0.808 (0.003)
		Ashing	20.91 (0.07)	0.334 (<0.001)	4.08 (0.02)	2.52 (<0.01)	32.88 (0.07)	0.811 (0.005) *
	Modified	Wet	28.37 (0.15)	0.499 (0.001)	4.12 (0.02)	2.68 (<0.01)	32.76 (0.07)	0.785 (0.004)
		Ashing	28.00 (0.08)	0.512 (0.002)	4.12 (0.02)	2.48 (<0.01)	33.58 (0.08)	0.871 (0.002) *
Crews	Standard	Wet	9.91 (0.10)	0.190 (<0.001)	0.04 (<0.01)	1.07 (<0.01)	19.74 (0.06)	0.828 (0.002)
		Ashing	9.96 (0.14)	0.192 (<0.001)	0.04 (<0.01)	1.09 (<0.01)	19.62 (0.05)	0.822 (0.004) *
	Modified	Wet	27.52 (0.13)	0.477 (0.001)	0.36 (<0.01)	2.12 (<0.01)	22.46 (0.08)	0.835 (0.003)
		Ashing	27.55 (0.13)	0.468 (0.001)	0.36 (<0.01)	2.16 (0.01)	22.98 (0.12)	0.820 (0.006) *

n = 3 for each treatment; * based on bioaccessibility estimated by difference.
